# The Senolytic Effect of Indole-3-Carbinol (I3C) on Mouse Embryonic (MEF) and Human Fibroblast Cell Lines

**DOI:** 10.3390/ijms252111652

**Published:** 2024-10-30

**Authors:** Scott L. Sax, Maria Laura Centomo, Federica Centofanti, Barbara Rizzacasa, Sierra Cox, Chelsea Cox, Andrea Latini, Maria Rosaria D’Apice, Liliana Mannucci, Giuseppe Novelli, Pier Paolo Pandolfi

**Affiliations:** 1 William N. Pennington Cancer Institute, Renown Health Nevada System of Higher Education, Reno, NV 89502, USA; ssax@unr.edu (S.L.S.); mlcentomo@mdanderson.org (M.L.C.); sierrac@nevada.unr.edu (S.C.); chelsea.cox@dri.edu (C.C.); 2 Desert Research Institute, Nevada System of Higher Education, Reno, NV 89502, USA; 3 Department of Biomedicine and Prevention, Tor Vergata University of Rome, 00133 Rome, Italy; federica.centofanti@uniroma2.it (F.C.); barbara.rizzacasa@uniroma2.it (B.R.); a.latini@med.uniroma2.it (A.L.); novelli@med.uniroma2.it (G.N.); 4 Medical Genetics Lab, Tor Vergata Hospital, 00133 Rome, Italy; d.apice@med.uniroma2.it (M.R.D.);; 5 Department of Pharmacology, School of Medicine, University of Nevada, Reno, NV 89557, USA

**Keywords:** senolytic, senescence, Indole-3-carbinol, apoptosis

## Abstract

Senescence and apoptosis are two fundamental cellular processes that play crucial roles in various physiological and pathological conditions. Senescence refers to the irreversible growth arrest that cells undergo in response to various stimuli, including telomeric alterations, stress, and oncogenic signaling. Pharmacological and/or genetic removal of senescent cells, also referred to as senolysis, triggers organ rejuvenation and tissue regeneration. Indole-3-carbinol (I3C) is a natural compound contained in *Brassicaceae* plants and identified in multiple in vitro and in vivo studies as a well-tolerated and effective compound in cancer prevention and therapy. Its anti-cancer properties have been attributed at least in part to its inhibitory activity of proto-oncogenic HECT E3-ubiquitin ligases such as NEDD4 and WWP1. While the tumor suppressive effects of I3C in cancer cell lines have been reported in multiple studies, little is known regarding the biological effects of I3C in primary normal cells, which attain spontaneous cellular senesce over serial passaging. To this end, we used two model systems: mouse embryonic fibroblasts (MEFs) and human primary dermal fibroblasts. Here, we surprisingly show that I3C does increase cellular senescence at early passages, while dramatically reducing the number of senescent cells through the induction of apoptosis in both mouse and human primary cells. Thus, our findings support the notion that I3C acts as a senolytic compound with important therapeutic implications for the prevention and treatment of aging manifestations. The notion can be readily tested in future clinical trials in humans also in view of the high tolerability and safety previously displayed by I3C in preclinical and clinical studies.

## 1. Introduction

Aging is a complex biological phenomenon that leads to a gradual loss of physiological functions and a predisposition to illness and death [1,2,3]. One of the main aging mechanisms is the accumulation of senescent cells in different tissues over time, which allows the development of several age-related diseases, like cardiovascular, neurodegenerative disorders, and cancer [1,4].

Although aging seems to be an antithetic process with respect to cancer, they share common biological processes: cellular damage and cellular senescence [2,5,6]. Cellular senescence is defined as a permanent state of cell cycle arrest, resistant to pro-apoptotic stimuli [7]. Several mechanisms, such as DNA damage, telomere dysfunction, epigenetic alterations, oncogene activation, altered intercellular communication, and organelle stress can trigger cellular senescence [2,8].

In 1961, Hayflick and Moorhead showed for the first time that normal human fibroblasts in culture can divide only a limited number of times before they stop growing, a state known as replicative senescence. This suggests that the aging of tissues may be due to the loss of cellular multiplication. The ability to proliferate is important for replacing damaged cells that build up over time [9]. If, on the one hand, senescence helps with tissue remodeling during development after injury, while halting the proliferation of DNA-damaged cells, on the other hand, it leads to inflammation, age-related tumorigenesis, and the progressive decline of tissue regenerative potential [10]. Several proteins, particularly cyclin-dependent kinase inhibitors (CDKIs), play pivotal roles in controlling cell cycle progression and maintaining cellular homeostasis. Two of them are the main drivers of the cell cycle arrest in senescence: p16^INK4a^ (encoded by the *CDKN2A* gene) and p21^CIP^ (encoded by the *CDKN1A* gene). p16 is a 136-kilobase protein that directly interacts with and inhibits CDK4/6, serving as a unique marker for senescence. Its transcriptional activation is widely used to indicate the presence of senescent cells in vivo, while its presence is also required to maintain the senescent state [10,11]. On the contrary, the p21 protein is mainly required for the induction of senescence and its expression does not persist in senescent cells. The 18-kDa p21 protein exhibits not only the ability to inhibit a spectrum of CDKs but is also critical for the induction of cell cycle arrest. However, p21 can be activated in a p53-independent manner, facilitated by pathways such as TNF-β and by employing Sp1 as the principal transcription factor, which makes it difficult to use it as a unique senescence marker [12].

Numerous studies have indicated that removing senescent cells from living organisms can enhance both lifespan and health span, just as it reduces age-related pathologies [13]. So, understanding cellular senescence’s mechanisms holds significant implications for multiple research fields, from aging research to cancer prevention and therapeutics.

Different strategies were developed to interfere with senescent cells and the consequences of their accumulation and are mostly based on two approaches: specific elimination of senescent cells and inhibition of components of the senescence-associated secretory phenotype (SASP). The first focuses on finding compounds that can specifically induce senescent cells to die (also defined as ‘senolytics’) and the last focuses on the inhibition of SASP without killing the senescent cell or reversing (defined as ‘senomorphics’) [14,15].

Many proteins and multiple pathways are involved in the suppression of cell death and can be potential targets for treatment. For example, the anti-apoptotic protein BCL-2 is increased in senescent cells, making them resistant to cell death, while the pro-apoptotic protein BAX is important in controlling cell death [16]. Senescent cells often have changes in BAX expression, which makes them resistant to cell death. Senolytic strategies aim to remove senescent cells by causing cell death, with BAX as a key target [7]. Additionally, the tumor suppressor protein p53, activated in senescent cells, leads to cell cycle arrest and the development of the senescence-associated secretory phenotype (SASP). Senolytic approaches can focus on p53-related pathways to eliminate senescent cells [17]. Developing senolytics may also aim at enhancing SIRT1 activity, a deacetylase that relies on NAD+ to regulate cellular aging and inflammation [18]. Another approach is to focus on TERT, aiming to eliminate senescent cells and promote health by maintaining telomere length and cellular immortality [19]. Chronic inflammation and age-related diseases are caused by TNF-α, a pro-inflammatory cytokine involved in the SASP in senescent cells. Senomorphics targeting TNF-α aim to reduce inflammation and help clear senescent cells [20].

Indole-3-carbinol (I3C) is a natural compound found in vegetables from the *Brassicaceae* family and has been extensively studied for its biological and pharmacological properties, including the suppression of cell cycle progression, the block of cancer cell migration, the induction of apoptosis, the inhibition of tumor growth, and SARS-CoV-2 viral egression [21]. These functions are at least in part the result of the I3C ability to enhance PTEN function through inhibition of the enzymatic activities of two proto-oncogenic enzymes of the HECT E3-ubiquitin ligases, NEDD4 and WWP1, and the consequent inhibition of PI3-Kinase and AKT signaling [22,23,24].

In addition, I3C displays cardioprotective, antioxidant, anti-inflammatory, antiangiogenetic, and antimicrobial properties [25]. Several studies highlight the ability of I3C to trigger apoptosis in different tumor cell lines [26,27,28,29]. Yoon DY’s group conducted a study that demonstrated how I3C leads to cell cycle arrest in G0/G1 and causes apoptosis via the Fas death receptor, triggering the activation of p-P53 and Caspase-8 in A549 lung carcinoma cells [30]. Another study reported that I3C had anti-leukemic properties in B cell precursor acute lymphoblastic leukemia (BCP-ALL NALM-6) cells, inducing cell growth inhibition by G1 cell cycle arrest and triggering apoptosis in a dose- and time-dependent manner [31].

While the tumor-suppressive effects of I3C in cancer cell lines have been reported in multiple studies, little is known regarding the biological effects of I3C in primary cells. To this end, we utilized two model systems: mouse embryonic fibroblasts (MEFs) and human primary dermal fibroblasts, two primary normal cells that attain spontaneous cellular senescence over serial passaging, and two widely recognized and extensively studied models in the context of senescence research. In this study, we subjected both primary cell lines to treatment with varying concentrations of I3C, monitoring their responses across multiple passages. Through a series of assays encompassing senescence markers, gene expression analyses, and morphological assessments, we aimed to elucidate the effects of I3C on cellular senescence pathways. Surprisingly, we demonstrated that I3C enhances cellular senescence at earlier passages, while at later passages, I3C treatment decreased the number of senescent cells by inducing apoptosis. Moreover, gene expression and protein analyses revealed a coordinated upregulation of senescence and apoptosis-associated markers upon I3C treatment, supporting its senolytic effects. Thus, our findings contribute to the understanding of senescence regulation and shed light on the therapeutic potential of dietary compound I3C in mitigating age-related pathologies.

## 2. Results

### 2.1. I3C Triggers the Removal of Senescent Cells Both in MEF and Human Fibroblast

#### 2.1.1. Effect of I3C on Senescence in MEF Cells

The leveraging of MEF cells is renowned for their susceptibility to senescence at early passages; we investigated how I3C influences the senescence processes in these cells.

**Figure 1:**
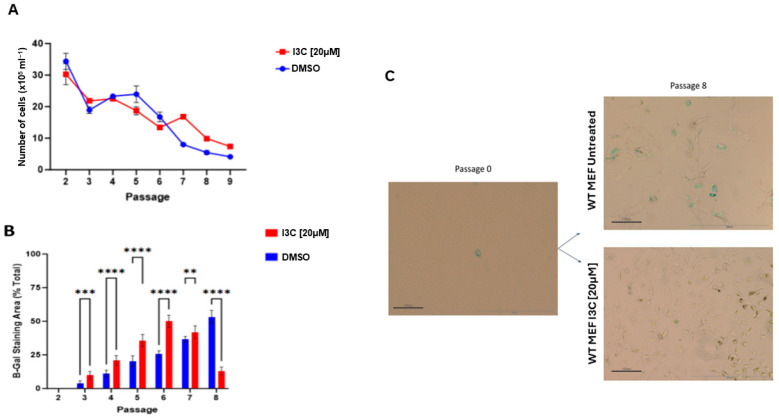
I3C treatment reduces the number of SA-β-Gal positive MEFs. (**A**) Graph of the total number of cells at each given passage for the treatment of MEF cells with I3C [20 µM] and untreated (DMSO-0.05%), evaluated in at least three independent experiments with 3 independent MEF cell lines; the results are represented as the mean ± SEM (*n* > 50). (**B**) Graph of the percentage of SA-β-Gal positive cells to the total number of cells was evaluated in at least three independent experiments, and the results are represented as the mean ± SEM (** *p* < 0.005; *** *p* < 0.001; **** *p* < 0.0001 by two-way ANOVA test). (**C**) Representative images of senescence-associated beta-galactosidase staining (SA-βGal) of normal MEF cells performed at passages 0 and 8 treated with I3C [20 µM] and no treatment. Scale bar 20×.

To observe senescence in MEFs, they are passed every 3 days until the ninth pass is reached. During this period, MEF cells were treated with I3C at 20 µM and DMSO-0.05% alone every 2 days to assess the effects of I3C on senescent cells. Cells were counted and stained at each passage for β-galactosidase to evaluate cell viability and the senescent cells (Figure 1).

The growth curve analyzed at each passage showed differences between the cells treated with I3C at 20 µM and the control cells (DMSO) (Figure 1A). In particular, from the fifth to the sixth passage, we observed a decrease in the number of cells treated with I3C [20 µM] compared to untreated cells; while from the sixth to the seventh passage, we observed an increase in the number of cells treated with I3C [20 µM] compared to untreated cells. Importantly, we analyzed the percentage of senescent cells by β-galactosidase assay and counted the number of senescent cells/Tot cells (Figure 1B,C). β-galactosidase revealed a significant increase in senescent cells treated with I3C at [20 µM] compared to untreated cells during the early passage (from the third to the sixth) (**** *p* < 0.0001). Surprisingly, we observed a switch from passage 7 to 8, where the percentage of β-gal-positive cells drastically decreases in MEF cells treated with I3C at 20 µM compared to untreated cells (**** *p* < 0.0001). These data suggested that I3C induces senescence while favoring their removal at later passages.

To understand if I3C could have senolytics properties, we conducted a comprehensive gene expression analysis in MEF cells at the eighth passage (the point at which we observed the reduction in senescent cells after I3C treatment). Specifically, we investigated genes associated with senolysis that are known to be influenced by I3C, including *BAX*, *BCL-2* (related to apoptosis), *TP53*, *P16INK4a*, *SIRT1*, *TERT* (senescence-associated), and *TNF-α* (pro-inflammatory cytokines) [32,33,34,35,36,37,38].

**Figure 2:**
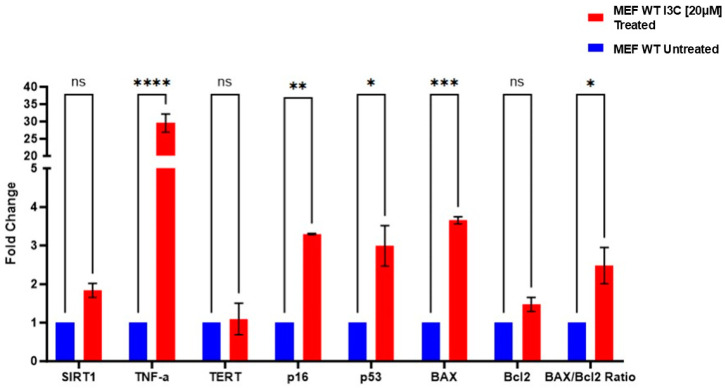
I3C treatment on the relative mRNA expression of targets relating to senolysis at 8th passages. Relative expression of *TP53*, *P16INK4a*, *BAX/BCL-2* ratio, *SIRT1*, *TERT*, and *TNF-α* mRNA levels in MEF cells treated with I3C [20 µM] and untreated at the eighth passage. The results are represented as the mean ± SEM (* *p* < 0.05; ** *p* < 0.01; *** *p* < 0.001; **** *p* < 0.0001; ns: not significant, *n* = 3).

Our analysis revealed a significant upregulation of *TP53*, *P16INK4a*, *BAX*, *BAX/Bcl2* ratio, and *TNF-α* mRNA levels within the treated group compared to the untreated group; no differences were observed in *SIRT1* and *TERT* gene expression (Figure 2).

We also evaluated the mRNA level of *TP53*, *P16INK4a*, and the *BAX/BCL-2 ratio* in each passage. We observed several fluctuations in these genes in the passages, indicating that the senescence pathway is complex and requires more in-depth research (Figure S1). However, this finding underscores the dynamic and time-dependent impact of I3C on the expression of genes crucial to age-related cellular processes. Furthermore, while the SA-β-Gal assay demonstrated a reduction in senescent cells following I3C treatment, the expression levels of senescent markers such as *P16INK4a*, *TP53*, *SIRT1*, and *TERT* appear to exhibit an opposing trend. This observation leads to suppose that I3C may act through a non-canonical pathway in contrast to conventional senolytic agents that suppress the expression of these markers.

**Figure 3:**
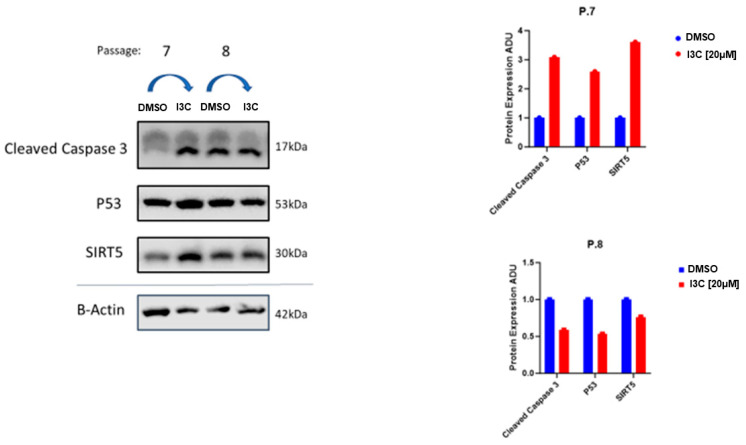
I3C treatment on the relative protein levels of targets relating to senolysis at 7th and 8th passages. Western blot and relative protein expression of Caspase 3, p53, and SIRT5 target proteins at passages 7 and 8 in MEF cells treated with I3C [20 µM] or DMSO. The results are normalized and quantified.

To assess the impact of I3C on protein expression levels, we performed Western blot analysis in passages 7 and 8. Specifically, we examined Cleaved Caspase-3, p53, and SIRT5 protein abundance at passages 7 and 8 due to the possibility of direct I3C targets [30] (Figure 3).

At passage 7, we observed a significant upregulation of Cleaved Caspase-3, p53, and SIRT5 protein levels in MEF cells treated with I3C at 20 µM compared to untreated MEF. On the other hand, in passage 8, we observed a significant downregulation of Cleaved Caspase-3, p53, and SIRT5 protein levels in MEF cells treated with I3C at 20 µM compared to untreated MEF.

#### 2.1.2. Effect of I3C on Senescence in Human Fibroblast Cells

To understand if I3C also had a senolytics effect on humans, we treated human dermal fibroblasts with 40 µM of I3C for 10 days and evaluated the effects. As mentioned before, the peculiarity of this cell line is that it goes against the normal senescence process as the passages increase [9].

**Figure 4:**
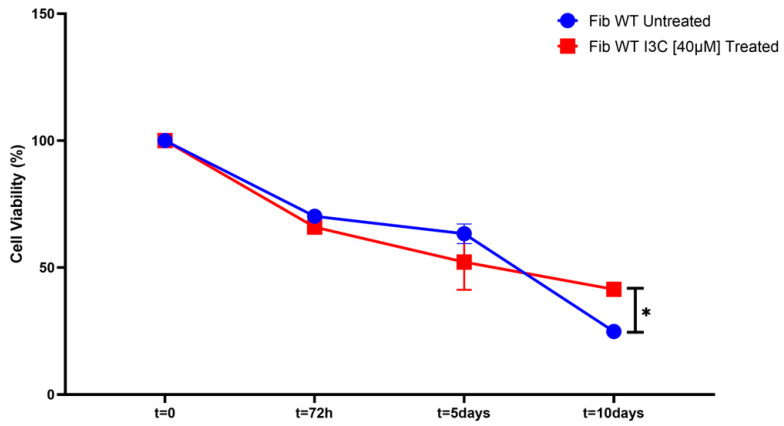
Effect of I3C on human fibroblast growth and cell viability. The graph shows the vitality of human fibroblast cells treated with I3C [40 µM] and left untreated (DMSO-0.05%) for 10 days. The total number of cells was evaluated in at least three independent experiments, and the results are represented as the mean ± SEM (* *p* < 0.05).

First, we evaluate cell viability in fibroblast cells treated and untreated with I3C [40 µM] using Trypan blue stain. After 72 h of treatment, we observed a decrease in cell viability of about 30–40% in both treated and untreated cells. This decrease in cell viability is even more evident in cells treated with I3C at day 5 of treatment than in untreated cells. Interestingly, upon continuing to treat cells with I3C [40 µM] for 10 days, there is a significant progressive increase in cell viability compared to untreated cells (* *p* < 0.02) (Figure 4).

**Figure 5:**
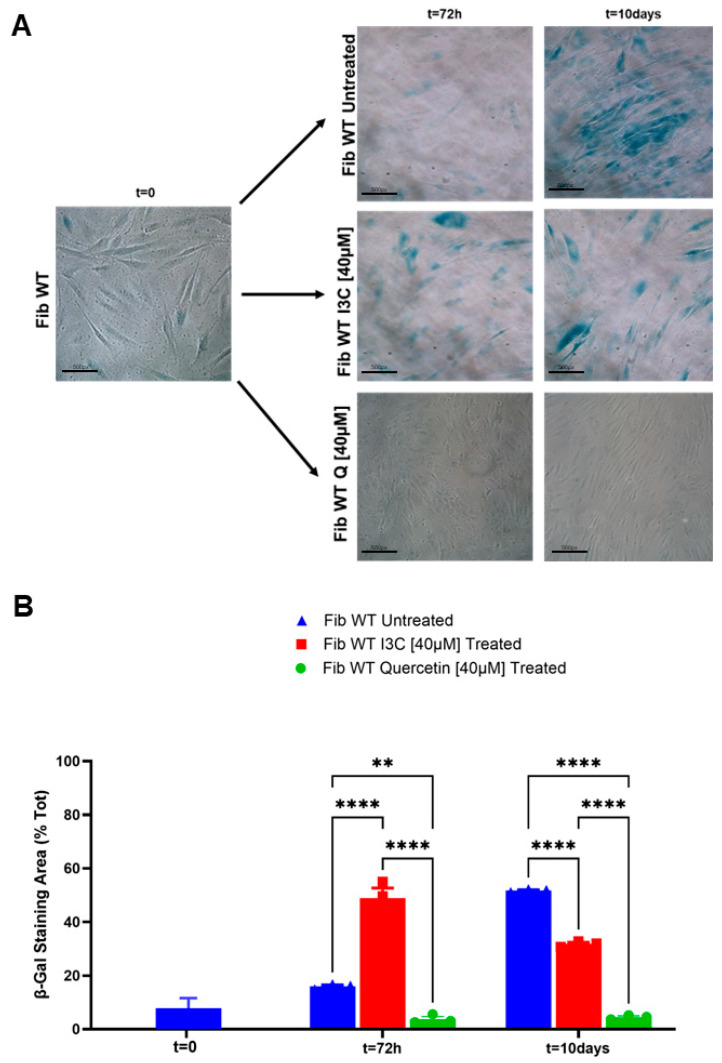
I3C and Quercetin treatment reduces the number of SA-β-Gal positive human fibroblast cells. (**A**) Representative images of senescence-associated β -Galactosidase staining (SA-β-Gal) of normal human dermal fibroblasts performed at 0, 72 h, and 10 days after being treated with I3C [40 µM], Quercetin [40 µM], and no treatment (Scale bar 20×). (**B**) Graph of the percentage of SA-β-Gal positive cells to the total number of cells was evaluated in at least three independent experiments, and the results are represented as the mean ± SEM (** *p* < 0.001; **** *p* < 0.0001 by one-way ANOVA test, *n* = 3). Blue bar: Fibroblast WT Untreated; red bar: Fibroblast WT Treated with I3C [40 µM]; green bar: Fibroblast WT Treated with Quercetin [40 µM].

Human fibroblast wild-type (WT) cells treated and untreated with I3C [40 µM] were stained for the β-Galactosidase assay and then counted as the number of senescent (blue) cells/Tot cells (Figure 5A). To compare the senolytic effect of I3C, we treated the cells in parallel with a well-known senolytic, Quercetin (Q). Quercetin displayed efficacy already after 72 h with just 3.1% senescent cells (**** *p* < 0.0001) detected, and its activity remains stable after 10 days (5.2%; **** *p* < 0.0001); while in the case of I3C, we observed completely different kinetics with a significant increase in senescent cells at 72 h in the I3C treated group [40 µM] compared to untreated cells (**** *p* < 0.0001). However, continuing to treat cells with I3C [40 µM] for 10 days, as before, we observed a significant decrease in senescent cells compared to untreated cells (**** *p* < 0.0001) (Figure 5B).

Once again, to deeply investigate the senolytic effects of I3C on human fibroblast, we performed a gene expression analysis with genes associated with senolysis, including *BAX*, *BCL-2*, and *Casp3* (related to apoptosis), *P16INK4a*, *P21CIP*, *TP53*, *SIRT1,* and *SIRT5* (senescence-associated), and *interleukin* (*IL*)*-6* and *TNF-α* (pro-inflammatory cytokines).

**Figure 6:**
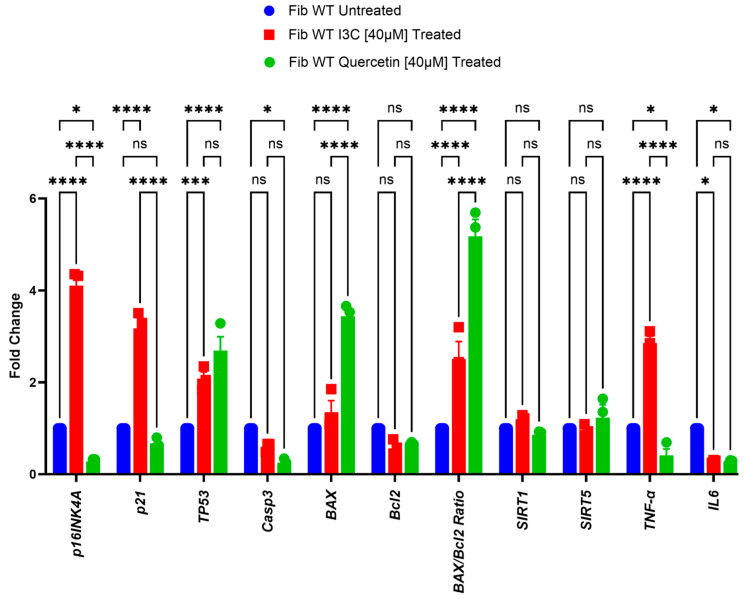
I3C and Quercetin treatment on the relative mRNA expression of targets relating to senolysis at 10 days. Relative mRNA expression of *BAX*, *BCL-2*, *Casp3*, *P16INK4a*, *P21CIP*, *TP53*, *SIRT1, SIRT5*, *IL-6* and *TNF-α* in human fibroblast cells treated with I3C [40 µM], Quercetin [40 µM], and no treatment. The bar graph shows the expression of genes relating to senolysis and cytokines quantified by qRT-PCR at 10 days of treatment. The results are represented as the mean ± SEM (* *p* < 0.05; *** *p* < 0.001; **** *p* < 0.0001; ns: not significant by one-way ANOVA test, *n* = 3). Blue bar: Fibroblast WT Untreated; red bar: Fibroblast WT Treated with I3C [40 µM]; green bar: Fibroblast WT Treated with Quercetin [40 µM].

After 10 days of I3C [40 µM] treatment, we observed a significant up-regulation in mRNA levels of *P16INK4a*, *P21CIP*, *TP53*, *TNF-α,* and the *BAX/Bcl2* ratio compared to an untreated fibroblast (*** *p* < 0.001; **** *p* < 0.0001); while we observed a significant downregulation in the mRNA level of *IL6* in fibroblasts treated with I3C [40 µM] compared to the control group (* *p* < 0.05). We did not observe any difference in *SIRT1*, *SIRT5*, *BAX*, *Bcl2,* and *Casp3* mRNA levels compared to untreated fibroblasts (Figure 6).

Unlike I3C, Quercetin triggered an opposite trend of senescence markers in agreement with the literature [39], i.e., *P16INK4a*, *TNF-α*, *IL6*, and *Casp3* showed a significant down-regulation in fibroblasts treated with Quercetin [40 µM] compared to the control group (* *p* < 0.05; **** *p* < 0.0001), while we observed a significant up-regulation in mRNA level of *TP53*, *BAX*, and the *BAX/Bcl2* ratio (**** *p* < 0.0001). By contrast, we did not observe any difference in *P21CIP*, *SIRT1*, *SIRT5,* and *Bcl2* mRNA levels compared to untreated fibroblasts.

**Figure 7:**
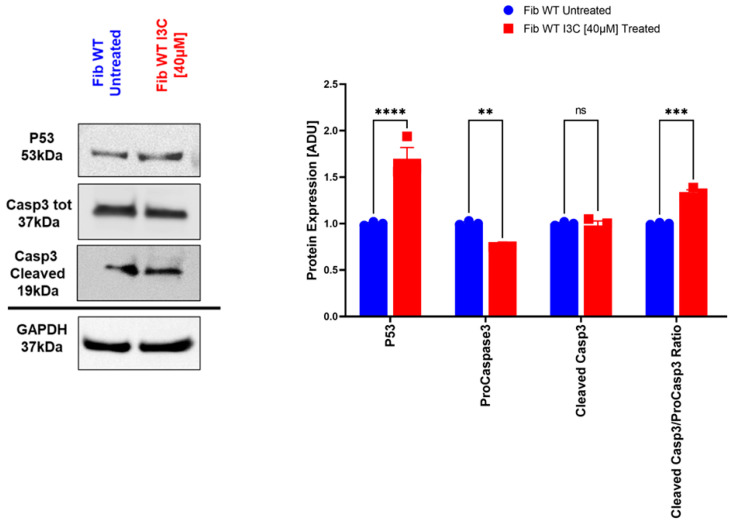
I3C treatment on the relative protein levels of targets relating to senolysis at 10 days. Blot and densitometric analysis of p53, ProCasp3, and Cleaved Casp3 in human fibroblast cells treated and untreated with I3C [40 µM]. The average was evaluated in at least three independent experiments and is reported as means ± SEM; (** *p* < 0.01; *** *p* < 0.001; **** *p* < 0.0001; ns: not significant estimated by a one-way ANOVA test, *n* = 3). Abbreviations: ADU, arbitrary densitometric units. Blue bar: Fibroblast WT Untreated; red bar: Fibroblast WT Treated with I3C [40 µM].

We also performed Western blot analysis to evaluate the protein abundance of p53 and Cleaved Casp3 proteins. After 10 days of I3C [40 µM] treatment, we observed a significant upregulation in protein levels of p53 and the Cleaved Casp3/Casp3 ratio (**** *p* < 0.0001 and ** *p* < 0.01, respectively) and a significant downregulation of ProCaspase3 protein levels (*** *p* < 0.001) in fibroblast treated with I3C [40 µM] compared to untreated fibroblasts. We did not observe any difference in Cleaved-Casp3 protein levels compared to untreated fibroblast (Figure 7).

## 3. Discussion

Cellular senescence, initially critical to halt tumorigenesis, can paradoxically promote cancer development later in life [40]. Additionally, the removal of senescent cells has become a potential treatment approach for preventing, delaying, or easing multiple diseases and age-related dysfunction. Encouraging results of senolytics in preclinical models indicate potential opportunities for therapy and prevention in delaying multiple diseases and increasing the period of good health [41].

Different therapeutic strategies take advantage of targeting vulnerabilities common to both cancer and senescent cells. Indeed, it has been demonstrated that the elimination of senescent cells with aging attenuates tumor formation in mice, raising the possibility that senolysis might be an effective strategy for preventing cancer [40]. Senolytic drugs may offer advantages over standard anticancer agents, as senescent cells do not proliferate and their numbers in tissues remain relatively low. Exploring drugs that disrupt pathways critical for cancer cell survival (for example resistance to apoptosis) may also hold promise as senolytic treatments [39,42]. To date, several strategies have been developed for the specific elimination of senescent cells. Among them, ABT263 and ABT737 are inhibitors of various members of the Bcl-2 family of antiapoptotic proteins; FOXO4-DRI, a peptide that forces the nuclear exclusion of p53; Quercetin, a polyphenol that inhibits PI3K; Dasatinib, an anticancer that inhibits EFNB1 and B3; and 2-deoxyglucose (2-DG) saturates the glycolytic flux as a false substrate for glucose hexokinase [43]. However, most of these drugs not only have intrinsic toxicities, but they target only a subset of senescence cells without discriminating between beneficial and harmful senescence programs, and this could limit their use for human purposes [10].

I3C and their derivates have demonstrated significant anti-cancer activity over the past 50 years. Multiple research articles have reported on their effectiveness, showing their potential as preventive and therapeutic agents against cancer. Specifically, I3C has been found to trigger apoptosis by influencing apoptosis markers and proteins in various cancer cell lines. Studies have also emphasized the potential benefits of I3C and DIM as promising anti-tumor options with minimal toxicity, as their antiproliferative effects are controlled by I3C-activated pathways [21,25].

Our data reveal new interesting findings regarding the effects of I3C treatment in primary cells. In both MEF and human fibroblasts, we observed an initial increase in cellular senescence with concomitant growth inhibition at early passages. Additionally, other senescence-related markers, such as *BAX/BCL-2* ratio, *TP53*, and *P16INK4a*, show similar trends in mRNA levels at early passages. This finding supports current data suggesting that I3C’s potential anti-cancer effects may be attributed to its initial induction of cellular senescence.

However, a dramatic switch occurs at passage 7, where, upon I3C treatment, the percentage of β-Gal-positive cells drastically decreases by passage 8. The same decrease in the percentage of β-Gal-positive cells is observed in human fibroblast after 10 days of I3C treatment. These results align with the observed senolytic properties of I3C at the molecular level. Indeed, in the treated group, we observed not only a significant upregulation of senescent pro-factors, including *TP53*, *P16INK4a*, *P21CIP*, *BAX/BCL-2* ratio, and *TNF-α* mRNA levels, but also a marked increase in Caspase-3, SIRT5, BAX/BCL-2, and p53 protein levels, in agreement with the observed induction of apoptosis.

Eukaryotic cell division is controlled by cyclin-dependent kinases (CDKs), which activate the phosphorylation of substrates and regulate the cell cycle [44]. Cell cycle arrest is initiated by the p53/p21WAF1/CIP1 and p16INK4A/pRB tumor suppressor pathways [45]. Prolonged stress activates p16INK4a, leading to cell cycle arrest by inhibiting CDK4/6 kinases [46]. The overexpression of p16INK4a is linked to both aging and senescence, while p53 regulates cell growth arrest, induction of cellular senescence, and/or induction of apoptosis [47]. Senescent cells take advantage of various molecular pathways to escape apoptosis, including the Bcl-2 protein family [48]. However, these markers are not exclusive to senescent cells and are not commonly used to ascertain this cell state. Instead, annexin V and cleaved caspase-3 are bona fide apoptotic markers [3]. Tumor necrosis factor (TNF) is a crucial cytokine in inflammatory reactions, and drugs that neutralize TNF are successful in treating chronic inflammatory and autoimmune diseases. TNF not only induces inflammatory gene expression but also triggers cell death and inflammatory immune reactions, contributing to disease development [49,50]. SIRT5, known for its role in regulating metabolic pathways and redox balance and maintaining mitochondrial and cellular homeostasis, has been found to influence both senescence and apoptosis [51]. Its increase at passage 7 and the subsequent increase in *BAX* mRNA at passage 8 support SIRT5’s role in promoting cell death in this context, while the increase in cleaved Caspase-3, p53, *BAX/BCL-2* ratio, and SIRT5 and the reduction in the percentage of β-Gal-positive cells indicate a clear shift toward apoptosis and senescence cells elimination.

Compared to other senolytic agents such as Quercetin, a drug able to induce apoptosis in senescent cells and to reduce markers of senescence (like *p16INK4A* and *p21CIP*) [12,39], I3C does seem to act differently, and further studies will be needed to investigate the molecular mechanism underlying its dual role as an inducer of senescence and senolysis. These first data, however, lead us to propose that I3C could be used in a two-step senescence-focused therapeutic concept that combines a pro-senescence induction followed by a senolytic phase that selectively eliminates senescent cells, with the advantage that I3C can be used as a single agent with such a dual action [52].

Collectively, our results support the senolytic properties of I3C, and our findings highlight the intricate roles of various factors, including SIRT5 and TNF-α, in the transition between cellular senescence and apoptosis. Understanding the intricate balance between these two cellular responses will be crucial for elucidating their roles in various physiological and pathological conditions, including cancer, aging, and tissue homeostasis. Our data also support the notion that I3C treatment may be beneficial to prevent tumorigenesis through the induction of cellular senescence but also by favoring the subsequent removal of senescence cells. This notion will need further validation in preclinical in vivo models and in clinical trials in humans, also in view of I3C limited toxicities [53,54,55].

## 4. Materials and Methods

### 4.1. Chemical Treatment

Indole-3-carbinol (I3C) was obtained from Sigma-Aldrich (Product Number: 17256, CAS-No.: 700-06-1). I3C was dissolved in 100% DMSO (Sigma-Aldrich, St. Louis, MO, USA) and added to the cell culture medium at [20 µM] and [40 µM], respectively.

Quercetin (Q) was obtained from Sigma-Aldrich (Product Number: Q4951, CAS-No.: 117-39-5). Q was dissolved in 100% DMSO (Sigma-Aldrich, St. Louis, MO, USA) and added to the cell culture medium at [40 µM] [1].

The controls were treated with the same low concentration of DMSO (<0.05%) as the treatment group to negate the effects of DMSO.

### 4.2. Cell Culture

MEF cells are mouse embryonic fibroblast cells originally extracted from the C57BL/6 mouse strain. These cells were maintained in DMEM (ThermoFisher Scientific, Waltham, MA, USA), supplemented with heat-inactivated 10% fetal bovine serum (FBS) (Gibco, Waltham, MA, USA), 1 mM L-glutamine (Gibco, Waltham, MA, USA), 1% penicillin/streptomycin (Gibco, Waltham, MA, USA) solution, and maintained at 5% CO_2_, 37 °C. Cells were detached and passaged using Trypsin (Gibco, Waltham, MA, USA) at a concentration of 0.05%.

Human fibroblast cells have been obtained from a dermal biopsy of a healthy donor (wild type, WT) already present in our database. Fibroblast cells were growing in DMEM (Sigma-Aldrich, St. Louis, MO, USA), supplemented with heat-inactivated 10% fetal bovine serum (FBS) (Gibco, Waltham, MA, USA), 1 mM L-glutamine (Gibco, Waltham, MA, USA), and 1% penicillin/streptomycin (ThermoFisher Scientific, Waltham, MA, USA) solution and maintained at 5% CO_2_, 37 °C.

### 4.3. Induction of Senescent Cells

MEF cells at an early passage (WT; passage 1) were seeded at a density of 17,000 cells/cm^2^ within a 10 cm culture dish, allowing them to adhere over 24 h. Subsequently, the culture medium was refreshed and the cells were subjected to treatment with a [20 µM] concentration of I3C, dissolved in a vehicle containing less than 0.05% DMSO, while the control group received the corresponding volume of DMSO alone. Media was replaced every 2 days, and these cells underwent regular passaging every 3 days, maintaining their exposure to the treatment throughout a total of 8 passages. At each passage interval, cellular samples were collected for comprehensive analysis.

Fibroblast wild-type (WT; passage 15) cells were seeded at a density of 7500 cells/cm^2^ in a 12-well plate and 20,000 cells/cm^2^ in a 6-well plate in DMEM-10%-FBS. After one day, the media was changed and the cells were treated with the proper amount of I3C [40 µM], Quercetin [40 µM], or 0.05% DMSO in DMEM-10%-FBS. The cells were treated for 10 days and then collected at 72 h and 10 days for further analysis.

### 4.4. Senescence-Associated β-Galactosidase Assay

A senescence-associated B-Gal assay was conducted following the SA-β-Gal kits protocol (ab65351). Cultured cells were washed once with PBS and fixed in SA-β-Galactosidase (SA-β-Gal) staining fix solution for 5 min at room temperature. Cells were washed thrice with PBS and incubated with SA-β-Gal staining solution with X-Gal [1 mg/mL] for 16 h at 37 °C. After the overnight incubation, cells were washed three times with PBS and observed under a bright field microscope (Nikon ECLIPSE TE2000-S, Nikon, Tokyo, Japan). The regions were randomly selected to be photographed to count the percentage of SA-β-Gal-positive cells/Tot cells using NIS-Elements F 5.22.00 software, Nikon, Tokyo, Japan). In total, 10 images at 20× were taken for each cohort with a minimum of 50 cells/image following [56]. SA-β-Galactosidase positive counts were performed by two trained investigators blinded to the experimental conditions.

### 4.5. Gene Expression

The total RNA was extracted from cells using Trizol Reagent (Invitrogen Life Technologies Corporation, Carlsbad, CA, USA), which was retro-transcribed and quantified relative to the gene expression levels, as reported in [53]. Primer sequences will be given upon request. The comparative ΔΔCt methods were used to quantify relative gene expression levels for three independent experiments.

### 4.6. Western Blotting

Cells were lysed in RIPA buffer (Thermofisher Waltham, MA, USA) containing protease and phosphatase inhibitor cocktails (Thermofisher, Waltham, MA, USA). After sonication and centrifugation at 13,000× *g* for 30 min at 4 °C, the supernatant was collected. Protein concentrations were determined by Bradford protein assay (Biorad, Hercules, CA, USA) and 50 µg of total cellular protein was separated by 4–12%SDS-PAGE (Thermofisher, Waltham, MA, USA). Proteins were then electroblotted onto nitrocellulose membranes (Thermofisher, Waltham, MA, USA) and were subsequently blocked with 5% non-fat dry milk in PBS-1X containing 0.1% (*v*/*v*) Tween-20 (Thermofisher, Waltham, MA, USA) for 1 h at room temperature and incubated with specific primary antibodies overnight at +4 °C: α-p53 (dil 1:300; Cell Signaling, Danvers, MA, USA), SIRT5 (D8C3; 1:1000; Cell Signaling, Danvers, MA, USA), Cleaved Caspase-3 (Asp175; 1:1000; Cell Signaling, Danvers, MA, USA), α-Casp3 (D3R6Y; 1:500; Cell Signaling, Danvers, MA, USA), and α-Pro-Casp3 (1:1000; Cell Signaling, Danvers, MA, USA). After three washes in PBS-T, the membranes were incubated with HRP-conjugated secondary antibodies. Proteins were then visualized with a chemiluminescence detection system Thermo Scientific™ SuperSignal™ West Pico PLUS Chemiluminescent Substrate (Thermofisher, Waltham, MA, USA) and the imaging system LAS-4000 mini (GE Healthcare, Chicago, IL, USA) or Odyssey XF (LICOR bio, Lincoln, NE, USA). Densitometric analysis was performed for three independent experiments using ImageJ software (bundled with 64-bit Java 8; https://imagej.net/ij/index.html) and monoclonal mouse α-GAPDH (1:2000; Thermofisher, Waltham, MA, USA) or β-Actin (1:2000; Thermofisher, Waltham, MA, USA) was used as normalized. For all SDS-PAGE, See Blue Plus 2 Pre-Stained Protein Ladder (3 to 198 kDa, Thermo Fisher Scientific) was used.

### 4.7. Cell Viability/Counts

Cell viability and counts have been assessed by trypan blue staining (Gibco, Waltham, MA, USA). Cells were collected every 72 h after treatment or after every passage and counted three times in either a Burker’s chamber as the percentage of cell death (blue)/Tot cells or using the cell drop (DeNovix, Wilmington, DE, USA).

### 4.8. Statistical Analyses

All the experiments were performed in technical triplicates and data were analyzed using GraphPad Prism 10.3.1 and SPSS version 19 (IBM Corp, Armonk, NY, USA). A paired Student t-test and one-way ANOVA test evaluated the difference between groups. The values displayed in the figures represent the means of three independent experiments ± standard error mean (SEM). Statistical significance was established at * *p* < 0.05, ** *p* < 0.01, *** *p* < 0.001, and **** *p* < 0.0001.

## Data Availability

The original contributions presented in the study are included in the article/Supplementary Material, further inquiries can be directed to the corresponding author.

## Supplementary Materials

The following supporting information can be downloaded at https://www.mdpi.com/article/10.3390/ijms252111652/s1.

## References

[1] Bientinesi E., Lulli M., Becatti M., Ristori S., Margheri F., Monti D. (2022). Doxorubicin-Induced Senescence in Normal Fibroblasts Promotes In Vitro Tumour Cell Growth and Invasiveness: The Role of Quercetin in Modulating These Processes. Mech. Ageing Dev..

[2] López-Otín C., Blasco M.A., Partridge L., Serrano M., Kroemer G. (2013). The Hallmarks of Aging. Cell.

[3] Bulbiankova D., Díaz-Puertas R., Álvarez-Martínez F.J., Herranz-López M., Barrajón-Catalán E., Micol V. (2023). Hallmarks and Biomarkers of Skin Senescence: An Updated Review of Skin Senotherapeutics. Antioxidants.

[4] Chaib S., Tchkonia T., Kirkland J.L. (2022). Cellular Senescence and Senolytics: The Path to the Clinic. Nat. Med..

[5] Montégut L., López-Otín C., Kroemer G. (2024). Aging and Cancer. Mol. Cancer.

[6] Sedrak M.S., Cohen H.J. (2023). The Aging-Cancer Cycle: Mechanisms and Opportunities for Intervention. J. Gerontol.—Ser. A Biol. Sci. Med. Sci..

[7] Hu L., Li H., Zi M., Li W., Liu J., Yang Y., Zhou D., Kong Q.P., Zhang Y., He Y. (2022). Why Senescent Cells Are Resistant to Apoptosis: An Insight for Senolytic Development. Front. Cell Dev. Biol..

[8] Di Micco R., Krizhanovsky V., Baker D., d’Adda di Fagagna F. (2021). Cellular Senescence in Ageing: From Mechanisms to Therapeutic Opportunities. Nat. Rev. Mol. Cell Biol..

[9] Hayflick L., Moorhead P.S. (1961). The Serial Cultivation of Human Diploid Cell Strains. Exp. Cell Res..

[10] Hernandez-Segura A., Nehme J., Demaria M. (2018). Hallmarks of Cellular Senescence. Trends Cell Biol..

[11] Baker D.J., Wijshake T., Tchkonia T., Lebrasseur N.K., Childs B.G., Van De Sluis B., Kirkland J.L., Van Deursen J.M. (2011). Clearance of P16 Ink4a-Positive Senescent Cells Delays Ageing-Associated Disorders. Nature.

[12] Du D., Tang X., Li Y., Gao Y., Chen R., Chen Q., Wen J., Wu T., Zhang Y., Lu H. (2022). Senotherapy Protects against Cisplatin-Induced Ovarian Injury by Removing Senescent Cells and Alleviating DNA Damage. Oxid. Med. Cell Longev..

[13] Childs B.G., Baker D.J., Wijshake T., Conover C.A., Campisi J., Van Deursen J.M. (2016). Senescent Intimal Foam Cells Are Deleterious at All Stages of Atherosclerosis. Science.

[14] Lozano-Torres B., Estepa-Fernández A., Rovira M., Orzáez M., Serrano M., Martínez-Máñez R., Sancenón F. (2019). The Chemistry of Senescence. Nat. Rev. Chem..

[15] Fuhrmann-Stroissnigg H., Ling Y.Y., Zhao J., McGowan S.J., Zhu Y., Brooks R.W., Grassi D., Gregg S.Q., Stripay J.L., Dorronsoro A. (2017). Identification of HSP90 Inhibitors as a Novel Class of Senolytics. Nat. Commun..

[16] Martin N., Popgeorgiev N., Ichim G., Bernard D. (2023). BCL-2 Proteins in Senescence: Beyond a Simple Target for Senolysis?. Nat. Rev. Mol. Cell Biol..

[17] Buffard T., Ferbeyre G., Muñoz-Espin D., Demaria M. (2020). Senolytics Target Senescent Cells and Improve Aging and Age-Related Diseases. Senolytics in Disease, Ageing and Longevity.

[18] Singh V., Ubaid S. (2020). Role of Silent Information Regulator 1 (SIRT1) in Regulating Oxidative Stress and Inflammation. Inflammation.

[19] Al-Naggar I.M.A., Kuchel G.A., Xu M. (2020). Senolytics: Targeting Senescent Cells for Age-Associated Diseases. Curr. Mol. Biol. Rep..

[20] Sun Y., Li Q., Kirkland J.L. (2022). Targeting Senescent Cells for a Healthier Longevity: The Roadmap for an Era of Global Aging. Life Med..

[21] Centofanti F., Buono A., Verboni M., Tomino C., Lucarini S., Duranti A., Pandolfi P.P., Novelli G. (2023). Synthetic Methodologies and Therapeutic Potential of Indole-3-Carbinol (I3C) and Its Derivatives. Pharmaceuticals.

[22] Kishikawa T., Higuchi H., Wang L., Panch N., Maymi V., Best S., Lee S., Notoya G., Toker A., Matesic L.E. (2021). WWP1 Inactivation Enhances Efficacy of PI3K Inhibitors While Suppressing Their Toxicities in Breast Cancer Models. J. Clin. Investig..

[23] Song M.S., Pandolfi P.P. (2022). The HECT Family of E3 Ubiquitin Ligases and PTEN. Semin. Cancer Biol..

[24] Lee Y.R., Chen M., Lee J.D., Zhang J., Lin S.Y., Fu T.M., Chen H., Ishikawa T., Chiang S.Y., Katon J. (2019). Reactivation of PTEN Tumor Suppressor for Cancer Treatment through Inhibition of a MYC-WWP1 Inhibitory Pathway. Science.

[25] Amarakoon D., Lee W.-J., Tamia G., Lee S.-H. (2023). The Annual Review of Food Science and Technology Is Online at Food. Annu. Rev. Food Sci. Technol..

[26] Rahman K.W. (2012). Novel Targets for Detection of Cancer and Their Modulation by Chemopreventive Natural Compounds. Front. Biosci..

[27] Ahmad A., A Sakr W., Wahidur Rahman K. (2010). Anticancer Properties of Indole Compounds: Mechanism of Apoptosis Induction and Role in Chemotherapy. Curr. Drug Targets.

[28] Firestone G.L., Sundar S.N. (2009). Minireview: Modulation of Hormone Receptor Signaling by Dietary Anticancer Indoles. Mol. Endocrinol..

[29] Firestone G.L., Bjeldanes L.F. (2003). Indole-3-Carbinol and 3-3′-Diindolylmethane Antiproliferative Signaling Pathways Control Cell-Cycle Gene Transcription in Human Breast Cancer Cells by Regulating Promoter-Sp1 Transcription Factor Interactions. J. Nutr..

[30] Choi H.S., Cho M.C., Lee H.G., Yoon D.Y. (2010). Indole-3-Carbinol Induces Apoptosis through P53 and Activation of Caspase-8 Pathway in Lung Cancer A549 Cells. Food Chem. Toxicol..

[31] Safa M., Tavasoli B., Manafi R., Kiani F., Kashiri M., Ebrahimi S., Kazemi A. (2015). Indole-3-Carbinol Suppresses NF-ΚB Activity and Stimulates the P53 Pathway in Pre-B Acute Lymphoblastic Leukemia Cells. Tumor Biol..

[32] Choi Y., Yanagawa Y., Kim S., Park T. (2013). Involvement of SIRT1-AMPK Signaling in the Protective Action of Indole-3-Carbinol against Hepatic Steatosis in Mice Fed a High-Fat Diet. J. Nutr. Biochem..

[33] Choi Y., Um S.J., Park T. (2013). Indole-3-Carbinol Directly Targets SIRT1 to Inhibit Adipocyte Differentiation. Int. J. Obes..

[34] Ware C.F., VanArsdale S., VanArsdale T.L. (1996). Apoptosis Mediated by the TNF-Related Cytokine and Receptor Families. J. Cell Biochem..

[35] Han M.K., Song E.K., Guo Y., Ou X., Mantel C., Broxmeyer H.E. (2008). SIRT1 Regulates Apoptosis and Nanog Expression in Mouse Embryonic Stem Cells by Controlling P53 Subcellular Localization. Cell Stem Cell.

[36] Lyn-Cook B.D., Mohammed S.I., Davis C., Word B., Haefele A., Wang H., Hammons G. (2010). Gender Differences in Gemcitabine (Gemzar) Efficacy in Cancer Cells: Effect of Indole-3-Carbinol. Anticancer. Res..

[37] CBrew C.T., Aronchik I., Hsu J.C., Sheen J.H., Dickson R.B., Bjeldanes L.F., Firestone G.L. (2006). Indole-3-Carbinol Activates the ATM Signaling Pathway Independent of DNA Damage to Stabilize P53 and Induce G1 Arrest of Human Mammary Epithelial Cells. Int. J. Cancer.

[38] Sarkar F.H., Rahman K.M.W., Li Y. (2003). Bax Translocation to Mitochondria Is an Important Event in Inducing Apoptotic Cell Death by Indole-3-Carbinol (I3C) Treatment of Breast Cancer Cells. J. Nutr..

[39] Zhang Z., Yang R., Zi Z., Liu B. (2024). A New Clinical Age of Aging Research. Trends in Endocrinology and Metabolism.

[40] Baker D.J., Childs B.G., Durik M., Wijers M.E., Sieben C.J., Zhong J., Saltness R.A., Jeganathan K.B., Verzosa G.C., Pezeshki A. (2016). Naturally Occurring P16 Ink4a-Positive Cells Shorten Healthy Lifespan. Nature.

[41] Huang W., Hickson L.T.J., Eirin A., Kirkland J.L., Lerman L.O. (2022). Cellular Senescence: The Good, the Bad and the Unknown. Nat. Rev. Nephrol..

[42] van Deursen J.M. (2019). Senolytic Therapies for Healthy Longevity. Science.

[43] Demirci D., Dayanc B., Mazi F.A., Senturk S. (2021). The Jekyll and Hyde of Cellular Senescence in Cancer. Cells.

[44] Malumbres M. (2014). Cyclin-Dependent Kinases. Genome Biol..

[45] Kanaki T., Makrantonaki E., Zouboulis C.C. (2016). Biomarkers of Skin Aging. Rev. Endocr. Metab. Disord..

[46] Kim W.Y., Sharpless N.E. (2006). The Regulation of INK4/ARF in Cancer and Aging. Cell.

[47] Kastenhuber E.R., Lowe S.W. (2017). Putting P53 in Context. Cell.

[48] Soto-Gamez A., Quax W.J., Demaria M. (2019). Regulation of Survival Networks in Senescent Cells: From Mechanisms to Interventions. J. Mol. Biol..

[49] Rath P.C., Aggarwal B.B. (1999). TNF-Induced Signaling in Apoptosis. J. Clin. Immunol..

[50] van Loo G., Bertrand M.J.M. (2023). Death by TNF: A Road to Inflammation. Nat. Rev. Immunol..

[51] Fabbrizi E., Fiorentino F., Carafa V., Altucci L., Mai A., Rotili D. (2023). Emerging Roles of SIRT5 in Metabolism, Cancer, and SARS-CoV-2 Infection. Cells.

[52] Bousset L., Gil J. (2022). Targeting Senescence as an Anticancer Therapy. Mol. Oncol..

[53] Centofanti F., Alonzi T., Latini A., Spitalieri P., Murdocca M., Chen X., Cui W., Shang Q., Goletti D., Shi Y. (2022). Indole-3-Carbinol in Vitro Antiviral Activity against SARS-CoV-2 Virus and in Vivo Toxicity. Cell Death Discov..

[54] Reed G.A., Arneson D.W., Putnam W.C., Smith H.J., Gray J.C., Sullivan D.K., Mayo M.S., Crowell J.A., Hurwitz A. (2006). Single-Dose and Multiple-Dose Administration of Indole-3-Carbinol to Women: Pharmacokinetics Based on 3,3′-Diindolylmethane. Cancer Epidemiol. Biomark. Prev..

[55] Fletcher A., Huang H., Yu L., Pham Q., Yu L., Wang T.T.Y. (2017). Reversible Toxic Effects of the Dietary Supplement Indole-3-Carbinol in an Immune Compromised Rodent Model: Intestine as the Main Target. J. Diet. Suppl..

[56] Krzystyniak A., Gluchowska A., Mosieniak G., Sikora E. (2023). Fiji-Based Tool for Rapid and Unbiased Analysis of SA-β-Gal Activity in Cultured Cells. Biomolecules.

