# Supplementary material for: The Senolytic Effect of Indole-3-Carbinol (I3C) on Mouse Embryonic (MEF) and Human Fibroblast Cell Lines
Source: Int J Mol Sci. 2024 Oct 30;25(21):11652. doi: 10.3390/ijms252111652 (PMC11546363; doi:10.3390/ijms252111652)
Supplement: Supplementary file 1 [file ijms-25-11652-s001.zip › ijms-3263393-supplementary-figure S1.pdf]

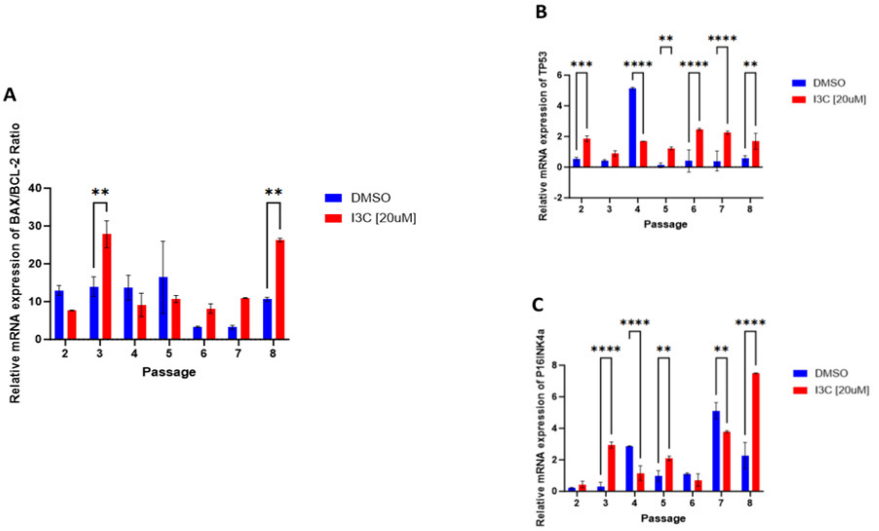

Figure S1. I3C treatment on the relative mRNA expression of targets relating to senolysis. A) Relative mRNA expression of the ratio of *BAX/BCL-2* of normal MEF cells performed from passages 2 to 8 treated with I3C [20µM] and untreated. The results are represented as the mean ± SEM (**p<0.01, n=3). **B)** Relative mRNA expression of *TP53* of normal MEF cells performed from passages 2 to 8 treated with I3C [20µM] and untreated. The results are represented as the mean ± SEM (**p<0.01; ****p<0.0001, n=3). **C)** Relative mRNA expression of *P16INK4a* of normal MEF cells performed from passages 2 to 8 treated with I3C [20µM] and untreated. The results are represented as the mean ± SEM (**p < 0.01; ****p<0.0001, n=3).
